# *RAD51*, *XRCC3*, and *XRCC2* mutation screening in Finnish breast cancer families

**DOI:** 10.1186/s40064-015-0880-3

**Published:** 2015-02-24

**Authors:** Liisa M Pelttari, Johanna I Kiiski, Salla Ranta, Sara Vilske, Carl Blomqvist, Kristiina Aittomäki, Heli Nevanlinna

**Affiliations:** Department of Obstetrics and Gynecology, University of Helsinki and Helsinki University Hospital, Biomedicum Helsinki, P.O. Box 700, FIN-00029 Helsinki, Finland; Department of Oncology, University of Helsinki and Helsinki University Hospital, P.O. Box 180, FIN-00029 Helsinki, Finland; Department of Clinical Genetics, University of Helsinki and Helsinki University Hospital, P.O. Box 160, FIN-00029 Helsinki, Finland

**Keywords:** Breast cancer, *RAD51*, *XRCC3*, *XRCC2*

## Abstract

Majority of the known breast cancer susceptibility genes have a role in DNA repair and the most important high-risk genes *BRCA1* and *BRCA2* are specifically involved in the homologous recombination repair (HRR) of DNA double-strand breaks. A central player in HRR is RAD51 that binds DNA at the damage site. The RAD51 paralogs RAD51B, RAD51C, RAD51D, XRCC2, and XRCC3 facilitate the binding of RAD51 to DNA. While germline mutations in *RAD51C* and *RAD51D* are associated with high ovarian cancer risk and *RAD51B* polymorphisms with breast cancer, the contribution of *RAD51, XRCC3,* and *XRCC2* is more unclear. To investigate the role of *RAD51, XRCC3,* and *XRCC2* in breast cancer predisposition and to identify putative recurrent founder mutations in the Finnish population where such mutations have been observed in most of the currently known susceptibility genes, we screened 182 familial Finnish breast or ovarian cancer patients for germline variation in the *RAD51*and *XRCC3* genes and 342 patients for variation in *XRCC2*, with a subset of the patients selected on the basis of decreased RAD51 protein expression on tumors. We also performed haplotype analyses for 1516 breast cancer cases and 1234 controls to assess the common variation in these genes. No pathogenic mutations were detected in any of the genes and the distribution of haplotypes was similar between cases and controls. Our results suggest that *RAD51, XRCC3,* and *XRCC2* do not substantially contribute to breast cancer predisposition in the Finnish population.

**Electronic supplementary material:**

The online version of this article (doi:10.1186/s40064-015-0880-3) contains supplementary material, which is available to authorized users.

## Introduction

Most of the known breast cancer susceptibility genes function in DNA damage repair. The most important predisposition genes *BRCA1* and *BRCA2*, conferring high life-time risks of breast and ovarian cancer, are involved in the homologous recombination repair (HRR) of DNA double-strand breaks (DSB) (Mavaddat et al. 2010). The moderate-penetrance genes *ATM*, *CHEK2*, *PALB2*, and *BRIP1* also have a role in DNA repair. A large proportion of the unexplained familial risk of breast cancer is likely explained by clustering of several common low-penetrance variants and so far, large number of low-risk loci have been identified (Michailidou et al. 2013). However, the currently known high, moderate, and low-penetrance alleles together only explain approximately 35% of the familial risk of breast cancer and thus, other susceptibility loci are likely to exist and genes involved in the homologous recombination repair are attractive candidates.

A central player in the homologous recombination is the RAD51 recombinase that binds to single-stranded DNA at break sites (Suwaki et al. 2011). The binding of RAD51 to DNA is facilitated by several proteins including BRCA2 and the five RAD51 paralogs RAD51B, RAD51C, RAD51D, XRCC2, and XRCC3. Deleterious germline mutations in the *RAD51C* and *RAD51D* genes confer an increased risk of ovarian cancer (Loveday et al. 2011, 2012) whereas common polymorphisms in the *RAD51B* gene are associated with male and female breast cancer (Figueroa et al. 2011; Orr et al. 2012). The contribution of *RAD51*, *XRCC3*, and *XRCC2* to breast cancer susceptibility remains unclear. Deleterious germline mutations in the *XRCC2* gene have been identified in exome sequencing of familial breast cancer patients but the association was not confirmed in a larger case–control study (Park et al. 2012; Hilbers et al. 2012). Several association studies of *XRCC3* have yielded controversial results yet a meta-analysis by He *et al*. suggests an association between common *XRCC3* polymorphisms and breast cancer risk (He et al. 2012). A likely deleterious missense mutation in the *XRCC3* gene has been identified in one breast and ovarian cancer family (Golmard et al. 2013). In the *RAD51* gene, one possibly disease-associated missense mutation has been identified in bilateral breast cancer patients whereas three studies report no deleterious *RAD51* mutations among breast cancer cases (Kato et al. 2000; Lose et al. 2006; Rapakko et al. 2006; Le Calvez-Kelm et al. 2012).

The presence of recurrent founder mutations in the Finnish population creates an advantage for the identification of new susceptibility genes. We have previously identified Finnish founder mutations in the ovarian cancer susceptibility genes *RAD51C* and *RAD51D* (Pelttari et al. 2011, 2012) and recently, we identified a recurrent nonsense mutation in the *FANCM* gene that associated especially with triple-negative breast cancer (Kiiski et al. 2014). In Finland, recurrent mutations explain most of the familial breast cancer risk caused by the currently known susceptibility genes, such as *BRCA1*, *BRCA2*, *PALB2*, and *CHEK2* (Sarantaus et al. 2000; Erkko et al. 2007; Vahteristo et al. 2002), whereas in other more diverse populations several rare mutations in each gene have been identified.

Inactivating mutations in tumor suppressor genes usually lead to decreased protein expression and further to tumor progression (Vogelstein and Kinzler 2004). Loss-of-function mutations have been identified in all the known breast cancer susceptibility genes involved in DNA damage response. We have previously shown that carriers of the truncating *CHEK2* c.1100delC mutation often have reduced or absent CHEK2 protein expression in breast tumors (Vahteristo et al. 2002). We have also previously identified two germline mutations in the *MRE11* gene among breast cancer patients whose tumors showed decreased expression of the MRN complex proteins MRE11, RAD50, and NBS1 that play an important role in the DNA damage response (Bartkova et al. 2008). The breast tumors were studied by immunohistochemical staining of MRE11, RAD50, and NBS1, and patients whose tumors had reduced expression of all three proteins were selected for further germline DNA analysis. These results indicate that loss or reduction of protein expression in the tumor may be a sign of underlying inactivating germline mutations.

To evaluate the contribution of *RAD51*, *XRCC3*, and *XRCC2* mutations to breast cancer predisposition, we screened 182 familial Finnish breast or ovarian cancer patients for germline variation in the *RAD51* and *XRCC3* genes and 342 patients for the *XRCC2* gene. To facilitate the mutation discovery, a subset of the patients was selected on the basis of decreased RAD51 protein expression on their breast tumors. We also studied the common variation in these genes with a haplotype analysis in 1516 breast cancer cases and 1234 controls.

## Materials and methods

### Subjects

The patient samples originated from two unselected series of breast cancer cases and additional familial breast and ovarian cancer patients collected at Helsinki University Hospital Departments of Oncology and Clinical Genetics (Eerola et al. 2000; Fagerholm et al. 2008). The unselected breast cancer cases were ascertained at Helsinki University Hospital Department of Oncology in 1997–1998 and 2000 (*n* = 884) (Syrjäkoski et al. 2000; Kilpivaara et al. 2005) and Department of Surgery in 2001–2004 (*n* = 986) (Fagerholm et al. 2008) including 79% and 87%, respectively, of all consecutive, newly diagnosed breast cancer cases during the collection periods. *BRCA1* and *BRCA2* mutation carriers were excluded from the familial patient series as previously described (Vahteristo et al. 2001, 2002; Vehmanen et al. 1997). RAD51 protein expression was analyzed in 1240 paraffin-embedded invasive breast tumors from these patients as described (Fagerholm et al. 2013).

The *RAD51* and *XRCC3* genes were screened in 182 and the *XRCC2* gene in 342 *BRCA1/2*-negative familial breast or ovarian cancer patients. Out of these, 71 were selected on the basis of absent or decreased RAD51 expression on tumors. The *RAD51*-*XRCC3* screening included two ovarian cancer probands and four cases affected with both breast and ovarian cancer and the *XRCC2* screening included five cases with breast and ovarian cancer; the rest of the screened patients were breast cancer cases. The patients had a strong family background of breast cancer with at least three breast or ovarian cancers among first or second degree relatives, including the proband. A haplotype analysis was performed in 1516 breast cancer cases (including 592 familial *BRCA1/2*-negative patients) and 1234 population controls that had been genotyped on the iCOGS chip (Michailidou et al. 2013). The population controls were healthy female blood donors from the same geographic region.

This study was performed with written informed consents from the patients and with permission from the Ethical review board of Helsinki University Hospital.

### Sequencing

The protein coding regions of the *RAD51, XRCC3,* and *XRCC2* genes were amplified by PCR in genomic DNA samples isolated from peripheral blood of the patients. The primers were designed with Primer3 software (http://bioinfo.ut.ee/primer3/). The PCR conditions are described in Additional file 1: Table S1. The PCR fragments were purified with ExoSAP-IT (Affymetrix) and subsequently sequenced using ABI BigDyeTerminator 3.1 Cycle Sequencing kit (Life Technologies). The capillary sequencing was performed at the Institute for Molecular Medicine Finland (FIMM), University of Helsinki, using 3730xl DNA Analyzer (Life Technologies). The sequence chromatograms were analyzed with FinchTV (Geospiza) and Variant Reporter software (Life Technologies).

### Bioinformatics and statistical methods

The pathogenicity of identified missense variants was evaluated with MutationTaster (Schwarz et al. 2010), SIFT, and PON-P (Olatubosun et al. 2012). The haplotype analysis was performed using PHASE v2.1.1 software (Stephens et al. 2001; Stephens and Scheet 2005) and the frequencies of haplotypes were compared between all breast cancer cases versus controls and familial breast cancer cases versus controls. The haplotypes were constructed using all single-nucleotide polymorphisms (SNPs) included in the iCOGS chip (Michailidou et al. 2013) that were located at the *RAD51* (*n* = 14), *XRCC3* (*n* = 10), and *XRCC2* (*n* = 10) gene loci and were not monomorphic in our study population. To test the association of the individual polymorphisms included in the haplotype analysis with breast cancer risk, two-sided p-values with odds ratios (OR) and 95% confidence intervals (CI) for each SNP were calculated using χ^2^ test or Fisher’s exact test when the count in any of the cells was five or less. Bonferroni’s adjustment was used for multiple-testing correction. We also studied the association of the missense mutations with 10-year breast cancer-specific survival using univariate Cox’s proportional regression models. The follow-up times were left-truncated at the date of ascertainment to account for the latency between diagnosis and study recruitment. The association analyses were performed using the R version 3.0.2 statistical software (http://www.r-project.org/).

## Results

In the sequencing of the *RAD51* gene, only intronic and untranslated region (UTR) variants were identified. In *XRCC3* and *XRCC2*, one known missense variant was identified in each gene (Table 1). Both missenses were predicted to be polymorphisms, tolerated, and neutral by MutationTaster, SIFT, and PON-P, respectively, and both were detected at comparable frequencies (31.3% for rs861539 in *XRCC3* and 4.7% for rs3218536 in *XRCC2*) as in the Finnish population of the 1000Genomes (31.7% for rs861539 and 4.8% for rs3218536) and of the Exome Aggregation Consortium (ExAC) (31.8% for rs861539 and 3.5% for rs3218536) (Exome Aggregation Consortium (ExAC), Cambridge, MA; http://exac.broadinstitute.org [January 2015]), and in the Sequencing Intiative Suomi (SISu) (30.1% for rs861539 and 3.9% for rs3218536) (http://sisu.fimm.fi/ [January 2015]) (Lim et al. 2014) dataset.

**Table 1:** **Identified germline variants in** ***RAD51, XRCC3,*** **and** ***XRCC2*** **genes**

**Gene**	**Genomic location** ^**a**^	**HGVS** ^**b**^	**Function**	**rs-number**	**AA** ^**c**^	**Aa** ^**d**^	**Aa** ^**e**^	**MAF** ^**f**^	**1000G-FIN MAF** ^**g**^
*RAD51*	15:40987528	c.-98G > C	5´UTR	rs1801320	154	27	1	0.080	0.113
*RAD51*	15:40987565	c.-61G > T	5´UTR	rs1801321	103	56	23	0.280	0.312
*RAD51*	15:40987568	c.-58C > G	5´UTR		181	1	0	0.003	
*RAD51*	15:40987725	c.-3 + 102C > T	intronic	rs3092981	151	22	9	0.110	0.183
*RAD51*	15:40991153	c.87 + 110A > G	intronic	rs2304579	153	28	1	0.082	0.113
*RAD51*	15:40998303	c.226-72delA	intronic	rs55943660	156	26	0	0.071	0.108
*RAD51*	15:40998342	c.226-33 T > G	intronic	rs45457497	136	43	3	0.135	0.129
*RAD51*	15:41001187	c.344-36 T > G	intronic	rs45455000	153	26	3	0.088	0.108
*RAD51*	15:41020898	c.531-12C > T	intronic		181	1	0	0.003	
*XRCC3*	14:104177282	c.55 + 88C > G	intronic		181	1	0	0.003	
*XRCC3*	14:104174944	c.108G > A	p.(=)		181	1	0	0.003	
*XRCC3*	14:104174824	c.193 + 34C > T	intronic	rs1799795	171	11	0	0.030	0.032
*XRCC3*	14:104173300	c.406 + 40C > T	intronic	rs374684710	177	5	0	0.014	
*XRCC3*	14:104169435	c.561 + 75G > A	intronic		181	1	0	0.003	
*XRCC3*	14:104165753	c.722C > T	p.(Thr241Met)	rs861539	91	68	23	0.313	0.317
*XRCC3*	14:104165647	c.774 + 54G > A	intronic	rs150986165	181	1	0	0.003	0.005
*XRCC3*	14:104165611	c.774 + 90G > T	intronic		181	1	0	0.003	
*XRCC3*	14:104165465	c.821 + 5G > A	intronic		181	1	0	0.003	
*XRCC3*	14:104165411	c.822-57C > T	intronic	rs17101777	181	1	0	0.003	
*XRCC3*	14:104165107	c.*28C > T	3'UTR		181	1	0	0.003	
*XRCC3*	14:104165100	c.*35A > G	3'UTR		181	1	0	0.003	
*XRCC2*	7:152373252	c.-88G > C	downstream	rs3218384	203	117	22	0.235	0.204
*XRCC2*	7:152373233	c.-69 T > G	5'UTR	rs3218385	324	16	2	0.029	0.032
*XRCC2*	7:152357877	c.40-10C > T	intronic	rs3218472	333	9	0	0.013	0.011
*XRCC2*	7:152346007	c.563G > A	p.(Arg188His)	rs3218536	310	32	0	0.047	0.048

^a^The genomic location is denoted according to NCBI37/Hg19 genome build and the variant coding refers to transcripts ENST00000267868 in *RAD51*, ENST00000352127 in *XRCC3*, and ENST00000359321 in *XRCC2*; ^b^variant description according to HGVS nomenclature; number of ^c^common homozygotes, ^d^heterozygotes, and ^e^rare homozygotes; ^f^minor allele frequency (MAF) observed in this study; ^g^MAF in 1000Genomes Finnish population.

The association of *RAD51, XRCC3,* and *XRCC2* haplotypes with breast cancer risk was studied among 1516 breast cancer cases (including 592 familial cases) and 1234 population controls. The haplotypes were constructed with PHASE v2.1.1 software using 14 polymorphic sites for *RAD51* and ten for *XRCC3* and *XRCC2*. Eleven *RAD51*, twelve *XRCC3*, and eight *XRCC2* haplotypes were predicted among the samples (Table 2). The distribution of the haplotypes did not differ between all the breast cancer cases and controls (*p* = 0.45, *p* = 0.49 and *p* = 0.55 for *RAD51, XRCC3,* and *XRCC2*, respectively) nor between the familial cases and controls (*p* = 0.66, *p* = 0.14 and *p* = 0.80 for *RAD51, XRCC3,* and *XRCC2*, respectively). We also tested the association of individual SNPs included in the haplotype analysis with breast cancer but none of them showed significant association (*p* = 0.060-0.951) (Table 3). After Bonferroni’s correction for multiple testing, *p*-value < 0.00167 was considered significant.

**Table 2:** **Detected haplotypes with frequency estimates for population controls and breast cancer cases**

***RAD51*** **haplotype**	**Haplotype count**	**Frequency controls**	**Frequency all cases**	**Haplotype count**	**Frequency controls**	**Frequency fam cases**
GCGCACTTAGAGAC	1510	26.66%	28.11%	999	26.66%	28.80%
GCGCATTTGGAGAC	1510	27.22%	27.63%	996	27.22%	27.36%
GCGTACTTAGGGAC	956	18.07%	16.81%	656	18.07%	17.74%
GTGCACTTAGAGAC	913	16.25%	16.89%	579	16.25%	15.03%
CCGCGCCGAAAGGG	431	8.51%	7.29%	301	8.51%	7.69%
GCTCATTTGGAGAC	138	2.76%	2.32%	97	2.76%	2.45%
GTGCACTTAGATAC	37	0.45%	0.86%	21	0.45%	0.84%
GCGCACTTAGGGAC	2	0.04%	0.03%	2	0.04%	0.08%
CCGCGCTTAGAGAC	1	0.04%	0%	1	0.04%	0%
GCGCACTTGGAGAC	1	0.001%	0.02%	0	0%	0%
CCGCGCCGAAAGAC	1	0%	0.03%	0	0%	0%
	**All BC cases vs controls:** ***p*** **= 0.45**			**Familial BC cases vs controls:** ***p*** **= 0.66**		
***XRCC3*** **haplotype**	**Haplotype count**	**Frequency controls**	**Frequency all cases**	**Haplotype count**	**Frequency controls**	**Frequency fam cases**
CATGCGCGGG	1599	28.57%	29.58%	1071	28.56%	31.07%
TACGCGCTGG	1586	28.32%	29.25%	1046	28.32%	29.30%
CGTACGCGGG	1159	22.33%	20.05%	778	22.33%	19.17%
CATGCGCGGA	602	10.82%	10.98%	378	10.83%	9.26%
CGTGCGTGGG	227	4.13%	4.03%	151	4.14%	3.98%
TACGCGCTAG	200	3.28%	3.92%	139	3.28%	4.90%
CGTGCGCGGG	75	1.49%	1.25%	55	1.49%	1.52%
CGTACACGGG	29	0.49%	0.56%	18	0.49%	0.51%
CGTGTGCGGG	17	0.41%	0.23%	12	0.41%	0.17%
CGTGCGTGGA	4	0.12%	0.11%	2	0.11%	0.03%
TGCGCGCTGG	1	0.05%	0.005%	1	0.05%	0.003%
CATGCGCTGG	1	0%	0.02%	1	0%	0.04%
	**All BC cases vs controls:** ***p*** **= 0.49**			**Familial BC cases vs controls:** ***p*** **= 0.14**		
***XRCC2*** **haplotype**	**Haplotype count**	**Frequency controls**	**Frequency all cases**	**Haplotype count**	**Frequency controls**	**Frequency fam cases**
GGGCGCACCT	3633	66.79%	65.48%	2438	66.80%	66.73%
GGGCGCACCG	1253	22.53%	22.97%	814	22.53%	21.79%
GGACGCACCT	247	4.13%	4.78%	159	4.13%	4.81%
GGGCCCATGT	123	2.15%	2.31%	84	2.15%	2.62%
AGGTGGACCT	123	2.25%	2.21%	83	2.25%	2.28%
GAGCGCGCGT	119	2.11%	2.21%	73	2.11%	1.77%
GGGCGCACGT	1	0.02%	0%	1	0.02%	0%
GAGCGCACCT	1	0%	0.02%	0	0%	0%
	**All BC cases vs controls:** ***p*** **= 0.55**			**Familial BC cases vs controls:** ***p*** **= 0.80**		

BC = breast cancer; Fam = familial.

Separate analyses were performed for all breast cancer cases versus controls and familial breast cancer cases versus controls. The SNPs included in the analysis are described in Table 3.

**Table 3:** **SNPs from the haplotype analysis with ORs and** ***p*** **-values for breast cancer association**

**Gene**	**rs-number**	**HGVS**	**MAF**_**controls**_	**MAF**_**cases**_	**OR**	**95% CI**	***p-*** **value**
*RAD51*	rs1801320	c.-98G > C	0.09	0.07	0.82	0.66-1.01	0.184
*RAD51*	rs3092981	c.-3 + 102C > T	0.17	0.18	1.07	0.91-1.27	0.614
*RAD51*	rs5030791	c.-3 + 203G > T	0.03	0.02	0.83	0.59-1.18	0.583
*RAD51*	rs2619681	c.-3 + 1398 T > C	0.18	0.17	0.88	0.75-1.05	0.352
*RAD51*	rs2304579	c.87 + 110A > G	0.09	0.07	0.82	0.67-1.01	0.184
*RAD51*	rs4924496	c.225 + 1936 T > C	0.29	0.29	1.06	0.90-1.24	0.549
*RAD51*	rs45503494	c.343 + 494 T > C	0.09	0.07	0.82	0.67-1.02	0.205
*RAD51*	rs45455000	c.344-36 T > G	0.09	0.07	0.82	0.67-1.02	0.202
*RAD51*	rs12592524	c.435 + 2149G > A	0.30	0.30	1.07	0.91-1.25	0.518
*RAD51*	rs4144242	c.436-4016G > A	0.09	0.07	0.82	0.67-1.02	0.202
*RAD51*	rs4924500	c.530 + 4654A > G	0.18	0.17	0.88	0.74-1.04	0.314
*RAD51*	rs45532539	c.531-3201G > T	0.004	0.009	1.92	0.97-4.10	0.062
*RAD51*	rs45507396	c.*929A > G	0.09	0.07	0.82	0.66-1.01	0.187
*RAD51*	rs45585734	c.*1113C > G	0.09	0.07	0.82	0.66-1.01	0.187
*XRCC3*	rs861539	c.722C > T	0.32	0.33	1.06	0.90-1.24	0.489
*XRCC3*	rs861537	c.562-1162G > A	0.29	0.26	0.89	0.76-1.04	0.060
*XRCC3*	rs861536	c.562-1651 T > C	0.32	0.33	1.06	0.90-1.24	0.475
*XRCC3*	rs12432907	c.561 + 1132G > A	0.23	0.21	0.92	0.78-1.08	0.092
*XRCC3*	rs3212092	c.561 + 866C > T	0.004	0.002	0.57	0.20-1.51	0.246
*XRCC3*	rs3212081	c.407-478G > A	0.005	0.006	1.15	0.55-2.49	0.704
*XRCC3*	rs3212079	c.407-801C > T	0.04	0.04	0.97	0.73-1.28	0.951
*XRCC3*	rs861531	c.406 + 533G > T	0.32	0.33	1.06	0.90-1.24	0.459
*XRCC3*	rs3212042	c.56-652G > A	0.03	0.04	1.17	0.86-1.58	0.456
*XRCC3*	rs3212028	c.-261 + 1368G > A	0.11	0.11	1.07	0.88-1.29	0.427
*XRCC2*	rs3218552	c.*1874G > A	0.02	0.02	0.95	0.66-1.37	0.879
*XRCC2*	rs3218550	c.*1772G > A	0.02	0.02	1.07	0.74-1.55	0.729
*XRCC2*	rs3218536	c.563G > A	0.04	0.05	1.08	0.82-1.43	0.256
*XRCC2*	rs3218504	c.122-4868C > T	0.02	0.02	0.94	0.65-1.36	0.878
*XRCC2*	rs6964582	c.122-5014G > C	0.02	0.02	1.02	0.70-1.47	0.676
*XRCC2*	rs3218501	c.122-5469C > G	0.02	0.02	0.93	0.64-1.34	0.839
*XRCC2*	rs3218491	c.121 + 4038A > G	0.02	0.02	1.04	0.72-1.52	0.817
*XRCC2*	rs3111465	c.40-4608 T > C	0.02	0.02	1.02	0.70-1.48	0.676
*XRCC2*	rs3094406	c.40-4998G > C	0.04	0.05	0.99	0.76-1.30	0.252
*XRCC2*	rs3218408	c.39 + 5510 T > G	0.23	0.23	1.09	0.93-1.28	0.447

The SNPs are presented in the same order as in the haplotypes in Table 2.

Since the *XRCC2* p.(Arg188His) variant (rs3218536) has been previously associated with poor breast cancer survival (Lin et al. 2011), we performed 10-year breast cancer-specific survival analyses for the *XRCC2* p.(Arg188His) missense variant as well as the *XRCC3* p.(Thr241Met) (rs861539) variant that were both detected in the sequencing of the genes and also included in the haplotype analysis. Patients with available follow-up information from the sequencing dataset and from the haplotype analysis (*n* = 1635, events = 106 for *XRCC2*; *n* = 1542, events = 80 for *XRCC3*) were combined for the survival analysis, including 1183 or 1176 cases from the unselected series and 452 or 366 additional familial cases for the *XRCC2* and *XRCC3* analysis, respectively. Given that most of the familial patients were prevalent cases with more than six months between breast cancer diagnosis and recruitment to the study, the data was left-truncated at the date of ascertainment. Neither of the missenses associated with breast cancer survival (hazard ratio (HR) = 0.67, 95% CI = 0.32-1.40, *p* = 0.288 for rs3218536; HR = 0.92, 95% CI = 0.66-1.29, *p* = 0.627 for rs861539).

## Discussion

We screened the *RAD51, XRCC3,* and *XRCC2* genes for germline variation in familial *BRCA1/2-*negative breast or ovarian cancer patients in order to evaluate the role of these genes in breast cancer predisposition in Finland and to identify putative recurrent founder mutations. To facilitate the variant discovery, we selected patients with strong family background of breast cancer from the homogeneous Finnish population where recurrent founder mutations in most of the breast cancer genes are present. In addition, a subset of the patients had decreased RAD51 protein expression on their breast tumors as we hypothesized that loss of protein expression might be a sign of underlying inactivating germline mutations. We also performed haplotype analyses in an extensive series of breast cancer cases and population controls to study the common variation in these genes.

No truncating mutations were identified in any of the genes. In *RAD51*, only intronic and UTR variants were identified which is in line with the previous studies where no cancer-predisposing mutations were identified among early-onset breast cancer patients (Lose et al. 2006; Rapakko et al. 2006; Le Calvez-Kelm et al. 2012). However, one of the detected 5’UTR polymorphisms, rs1801320, has been found to affect the splicing of *RAD51* within the 5’UTR and to modify breast cancer risk among *BRCA2* mutation carriers (Levy-Lahad et al. 2001; Antoniou et al. 2007). In *XRCC3* and *XRCC2*, known missense variants p.(Thr241Met) and p.(Arg188His), respectively, were detected. Both of these variants were detected at comparable frequencies as in the Finnish population of the 1000Genomes, ExAC, and SISu datasets and neither was predicted to be pathogenic *in silico*. A large association study by Breast Cancer Association Consortium (BCAC) found no association with breast cancer risk for neither of the variants (Breast Cancer Association Consortium 2006). However, a meta-analysis by He *et al*., including also the BCAC study, suggests the *XRCC3* p.(Thr241Met) variant is associated with a mild increase in breast cancer risk (OR = 1.10, 95% CI = 1.03-1.16) (He et al. 2012). In our data set, the p.(Thr241Met) and p.(Arg188His) variants did not associate with an increased breast cancer risk nor did they form risk-associated haplotypes. Furthermore, the overall distribution of *RAD51, XRCC3,* or *XRCC2* haplotypes did not differ between all breast cancer cases and controls or between familial cases and controls. Previously, *XRCC2* p.(Arg188His) has been associated with poor breast cancer survival (Lin et al. 2011) but in our study, no survival effect was found for this variant or for the *XRCC3* p.(Thr241Met) variant.

Like most of the known breast cancer susceptibility genes, *RAD51, XRCC3,* and *XRCC2* also have a role in DNA double-strand break repair by homologous recombination. XRCC2 and XRCC3, two of the five human RAD51 paralogs, help to load RAD51 on the site of DNA damage (Suwaki et al. 2011). *XRCC2* gene has been recently linked to breast cancer since rare germline mutations in the gene were identified in breast cancer families (Park et al. 2012). However, no association with breast cancer risk was detected in a subsequent large case–control study (Hilbers et al. 2012) and another study by Golmard *et al.* (Golmard et al. 2013) reports no pathogenic *XRCC2* mutations among early-onset or familial breast cancer patients (Golmard et al. 2013). Interestingly, one Fanconi anemia patient has been found to carry a homozygous truncating *XRCC2* mutation (Shamseldin et al. 2012) while biallelic mutations in four breast and ovarian cancer susceptibility genes, *BRCA2*, *BRIP1*, *PALB2*, and *RAD51C*, are associated with Fanconi anemia (Kee and D'Andrea 2012). Given the unclear role of *XRCC2* in breast cancer susceptibility, we sequenced the gene in an extensive series of 342 patients with a strong family history breast cancer. As the only identified coding variant was a neutral missense mutation, our results indicate that *XRCC2* is not a major breast cancer susceptibility gene, in line with the studies by Hilbers *et al*. and Golmard *et al*. In contrast to *XRCC2*, no truncating mutations in *XRCC3* or *RAD51* genes have been reported and only one possibly disease-associated missense in each gene has been detected in breast cancer patients (Golmard et al. 2013; Kato et al. 2000). Furthermore, the *RAD51* missense mutation was later also detected once among 1330 breasts cancer cases as well as once among 1123 controls (Le Calvez-Kelm et al. 2012). The absence of mutations in our study as well as the results of the previous studies indicates that *XRCC3* and *RAD51* are not major breast cancer susceptibility genes.

## Conclusions

In conclusion, the absence of mutations among breast cancer families and similar distribution of haplotypes between breast cancer cases and controls suggests that *RAD51, XRCC3,* and *XRCC2* do not substantially contribute to familial breast cancer predisposition in the Finnish population. Taken together, it is unlikely that *RAD51, XRCC3,* and *XRCC2* have a significant contribution to breast cancer susceptibility. However, we cannot exclude possible unique or very rare risk variants.

## Additional file

Additional file 1: Table S1. Primers and PCR conditions for the sequencing of the *RAD51*, *XRCC3*, and *XRCC2* genes.
