# Supplementary material for: RAD51, XRCC3, and XRCC2 mutation screening in Finnish breast cancer families
Source: Springerplus. 2015 Feb 24;4:92. doi: 10.1186/s40064-015-0880-3 (PMC4404470; doi:10.1186/s40064-015-0880-3)
Supplement: Additional file 1: Table S1. — Primers and PCR conditions for the sequencing of the RAD51, XRCC3, and XRCC2 genes. [file 40064_2015_880_MOESM1_ESM.pdf]

RAD51, *XRCC3*, and *XRCC2* mutation screening in Finnish breast cancer families

Liisa M. Pelttari, Johanna I. Kiiski, Salla Ranta, Sara Vilske, Carl Blomqvist, Kristiina Aittomäki, Heli Nevanlinna

Corresponding author: Heli Nevanlinna, Department of Obstetrics and Gynecology, University of Helsinki and Helsinki University Hospital, heli.nevanlinna@hus.fi

Supplementary Material

Supplementary Table 1. Primers and PCR conditions for the sequencing of the *RAD51*, *XRCC3*, and *XRCC2* genes.

Gene	Amplicon	Length (bp)	Forward primer	Reverse primer	Temp (C°)
RAD51	5'UTR-ex1	540	TTACGCAAAAAGGGAAGAGG	GTCCGGGTTTCACACTGC	58
RAD51	ex2	443	GGCCCCTACACTGAAGGAAT	GGCCCTGCCAGACATATTTA	55
RAD51	ex3	398	CCAAGGATTTCAAGGGACAG	GGTAACAGTCACCCCTGGAA	55
RAD51	ex4	526	CCCATTGCACACCTTGTTTT	TGCACCTACAGGGATTACAGG	55
RAD51	ex5	288	ATCGCTTGTTGGAAATTGAA	GATGCTTGCAAATAGCAGGA	55
RAD51	ex6	274	TGTCATGAGGAGCTTGGTCA	TTGGGACGTGGGGTTATAGA	58
RAD51	ex7	370	TGGGTGCTATCATCTCTCTGC	GCCATTACAAGATTCAGGGAAG	58
RAD51	ex8	253	TGGTAAGGAAGGGACCAGAA	CTGGAATTCACAGCCACTGA	58
RAD51	ex9	421	GCAGCATTAAGGCTTTTGGA	ACAGGGGAGAGGCATATCAA	58
RAD51	ex10	416	AAATGGATTCTGCCAGGTTG	TTTCCCGGAAGCTTTATCCT	58
XRCC3	ex3	495	GGTAAATGTGGTGGGTTTGG	CGCCCTCGGTTTAATAATCA	58
XRCC3	ex4	507	CACACTGTGTCCAGGGTTTC	ATCTTCTGACCCGATGCTGT	60
XRCC3	ex5	467	CTTGCTCACCCCCATGAC	AATGGTAGGAACAGCGCAAG	61
XRCC3	ex6	345	GTTAGAAATGGCGGGAGACA	CTGCTGTGAGACCCCATTG	61
XRCC3	ex7	423	GACAGTCCAAACGGGGTCT	GGTTAGGCACAGGCTGCTAC	65
XRCC3	ex8-9	531	GTAGCAGCCTGTGCCTAACC	CCAGGCAGACGCGTTTTA	61
XRCC2	ex1	249	AGGCCCGCACACCCTATT	GGCCTTGTTCCCATCTCC	63
XRCC2	ex2	378	ACAGCACCCAGCCTAAAGTT	TTGTCTGACACCGATGAGGA	63
XRCC2	ex3a	485	TGCATTTTGTAACCTACCCTTG	TCTCTAAGCACTGAGAACATTTCC	61
XRCC2	ex3b	528	TCAATGGAGGAGAAAGTGTGAA	AGGAGGCACACATAGGAGGA	60

Bp = base pairs, Temp = annealing temperature.
